# Coupling of Li–Fe: Li Isotope Fractionation during Sorption onto Fe-Oxides

**DOI:** 10.1021/acsearthspacechem.4c00205

**Published:** 2024-11-25

**Authors:** Xu Yvon Zhang, David J. Wilson, Maartje F. Hamers, Philip A. E. Pogge von Strandmann, Josephina J. P. A. Mulders, Oliver Plümper, Helen E. King

**Affiliations:** † Department of Earth Sciences, Utrecht University, 3584 CB Utrecht, The Netherlands; ‡ LOGIC, Department of Earth Sciences, University College London, WC1E 6BS London, U.K.; § MIGHTY, Institute for Geosciences, Johannes Gutenberg University Mainz, D-55128 Mainz, Germany; ⊥ Evides Water, Schaardijk 150, 3063 NH Rotterdam, The Netherlands

**Keywords:** lithium, weathering, water–rock interaction, iron oxides, crystallinity

## Abstract

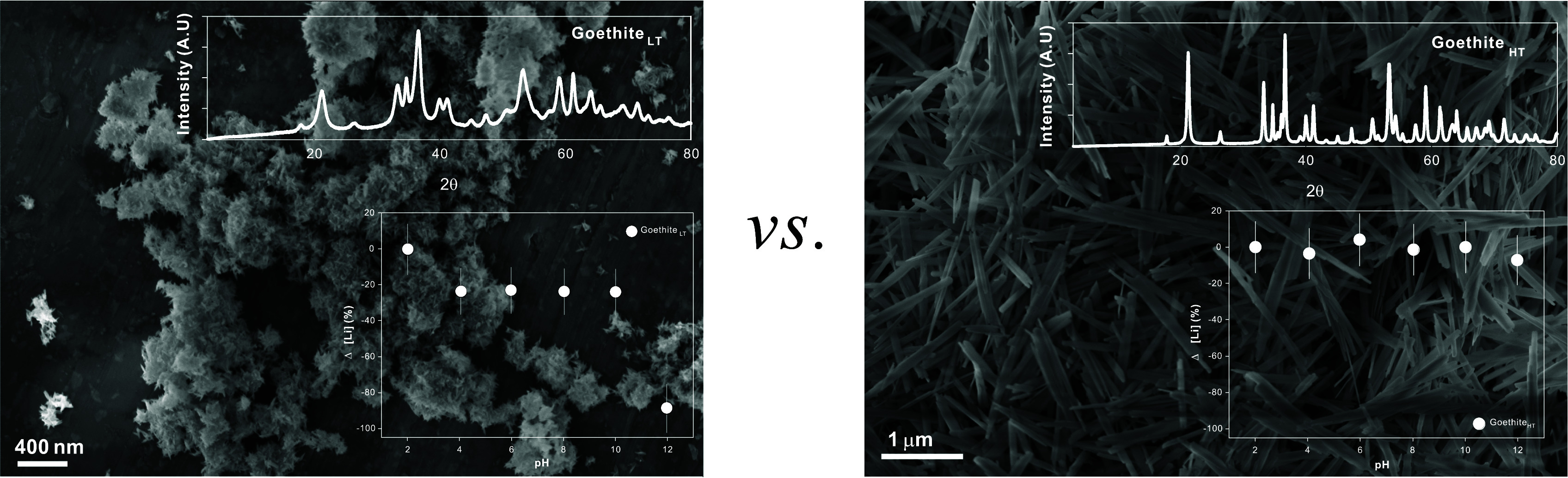

Chemical weathering processes play a key role in regulating the global climate over geological time scales. Lithium (Li) isotope compositions have proven to be a robust proxy for tracing weathering processes that produce secondary minerals, such as clays and oxides, with a focus often placed on Li adsorption to, or incorporation into, clay minerals. In addition, the interaction between Li and Fe-oxides has long been assumed and discussed based on field observations, but experimental constraints on this process are lacking. Here, we investigated the geochemical behavior of Li during its sorption onto individual Fe-oxides, including goethite, hematite, wüstite, and magnetite. With a point of zero charge at ∼7.7, poorly crystallized goethite nanoparticles take up ∼20% of dissolved Li over a pH range from ∼4 to ∼10, rising to ∼90% at pH ∼12. In contrast, the sorption of dissolved Li is insignificant for well-crystallized Fe-oxides (hematite, wüstite, magnetite, and goethite). This Li uptake by poorly crystallized goethite is likely attributed to dissolution and reprecipitation reactions at poorly crystalline goethite surfaces. The goethite particles preferentially take up light ^6^Li isotopes, resulting in an isotope fractionation of Δ^7^Li_oxide-fluid_ ∼ −16.7 to −20.1‰. Overall, our study provides valuable data to better understand the processes occurring in highly weathered soil and sediment profiles that are rich in Fe-oxides, such as laterites. This research also emphasizes the significance of chemistry at mineral surfaces during mineral–water interactions and illuminates the mechanisms of large-scale Li extraction for future applications.

## 1 Introduction

The complex interactions among the lithosphere, atmosphere, hydrosphere, and biosphere at the Earth’s surface play a crucial role in shaping landscapes, in facilitating the transfer of matter from the continents to the oceans, and in regulating long-term climate via the consumption of atmospheric carbon dioxide through silicate weathering.^1,2^ Using lithium (Li) isotopes to investigate and quantify water–rock interactions has been shown to be a robust approach due to (1) an enrichment of Li in secondary phases such as clays; (2) significant Li isotope fractionation due to the large relative mass difference between the two isotopes (^7^Li and ^6^Li); and (3) little involvement of Li in biological processes.^3−8^

In the past three decades, there have been major advances in our understanding of Li isotope geochemistry, which has received considerable attention due to its applications for water–rock interactions and weathering studies. During water–rock interactions, the lighter ^6^Li is preferentially retained in solid secondary materials, causing an enrichment of heavy ^7^Li in the fluid phase.^9−12^ Therefore, Li isotope compositions in geological materials (expressed as δ^7^Li values, in permille relative to the ^7^Li/^6^Li ratio in the standard reference material L-SVEC: δ^7^Li (‰) = ((^7^Li/^6^Li)/(^7^Li/^6^Li)_L-SVEC_ – 1) × 1000) can be used to investigate chemical weathering histories,^5,13−23^ seawater-composition evolution,^4,24−26^ and authigenic clay formation.^26−28^

A number of experimental approaches have been used to constrain Li isotope fractionation during water–rock interactions under both high-temperature^29−33^ and low-temperature^9−12,34−37^ conditions. In general, these studies demonstrate that (1) there is little Li isotope fractionation associated with mineral dissolution processes; (2) Li isotope fractionation takes place during the formation of secondary phases; and (3) Li isotope fractionation is inversely related to temperature, with less fractionation at high temperatures.

Despite these significant advancements in Li isotope geochemistry, a few key scientific questions remain under-addressed. In particular, how Li behaves during interactions between Fe-(oxyhydr)oxides and solutions requires further investigation.^38^ Iron oxide minerals are the dominant constituents of laterite and lateritic soils, which represent the products of prolonged and/or intense weathering processes.^39,40^ Observations on such deposits show a more complex relationship between Li behavior (Li concentrations and δ^7^Li values) and Fe-oxide-rich materials compared to the Li behavior in systems dominated by silicates. For example, in a laterite profile from Deccan, India, Kısakürek et al.^41^ observed a distinct difference in Li behavior between a paleo-watertable sample with highly elevated Fe contents, which had low Li concentrations and low δ^7^Li values, and other samples from the same depth profile with lower Fe contents that were characterized by a negative relationship between Li concentrations and Li isotopes. The authors attributed the former observation to a weathering signal and the latter observation to an external dust endmember mixing with the laterite materials. In laterite soil profiles from Yunnan, China, Ji et al.^42^ reported a negative correlation between Si isotopes and Li isotopes and a positive relationship between Li isotopes and Fe^3+^/Fe^2+^ ratios. These observations are intriguing because they also differ from findings for phyllosilicate-rich materials, in which Si isotopes and Li isotopes are positively correlated.^28,43^ In addition, the correlation between δ^7^Li values and Fe^3+^/Fe^2+^ ratios in the laterite seemingly implies that redox conditions may play a role in setting Li isotope signatures in oxides, even though Li has only one valence state. Unfortunately, in these studies, no oxides were separated from the bulk soils for analysis. Therefore, the observed relationships between Li and Fe content remain empirical, and the mechanisms driving the coupling between Li and Fe are unclear.

In non-laterite profiles, Fe-oxides have also been proposed to modify fluid Li geochemistry.^21,44,45^ For example, in Iceland, ferrihydrite is one of the most common secondary phases formed during the weathering of basalts and is found even in young soils, where interesting relationships between the Fe content and the δ^7^Li values of both solids and solutions have also been observed.^46,47^ On the one hand, it has been suggested that the formation and presence of ferrihydrite could potentially fractionate Li isotopes and generate high δ^7^Li values in the fluids.^46^ However, no correlation is observed between the abundance of ferrihydrite and the δ^7^Li signature in Icelandic soils.^47^

How Li interacts with Fe-oxides remains insufficiently addressed due to a lack of experimental investigations. For example, experimental work has shown that different Li isotope fractionations can be associated with Li uptake by the various locations (octahedral site, outer-sphere complex, etc.) of clay minerals.^9,10^ In contrast, it is unclear if similar mechanisms are in operation during the interaction between Li and Fe-oxides, and the literature presents contradictory suggestions for the association of Li with Fe-oxides during water–rock interactions.^48,49^ On the one hand, studies of suspended sediments from Greenland and a catchment observatory in Shale Hills (Pennsylvania, USA) assumed that a significant amount of Li may be taken up by Fe-(oxyhydr)oxides^44,48^ and suggested an associated isotope fractionation of ∼−20‰.^48^ On the other hand, the Li geochemistry of marine ferromanganese deposits^49^ implies that little seawater Li is adsorbed onto the surface of goethite or amorphous FeOOH, which hold a slightly positive charge at seawater pH values.

To date, only one study has directly investigated the effect of Fe-oxides on fluid Li geochemistry. A single experiment conducted by Pistiner and Henderson^34^ has shown that a moderate proportion of dissolved Li (32%) can be taken up by ferrihydrite after 24 h, generating a change in fluid δ^7^Li values of 1.6‰. The associated Li isotope fractionation (Δ^7^Li_solid–fluid_ = δ^7^Li_solid_ – δ^7^Li_fluid_ = ∼−3.5‰) is significantly smaller than the fractionation observed during clay formation, which typically ranges from −16 to −22‰,^9−12,50,51^ but is close to some fractionations observed for Li adsorption onto exchangeable outer-sphere sites of clay minerals (Δ^7^Li ∼ 0‰).^9,10,34^ In contrast, indirect approaches based on oxide leaching methods suggest a larger Li isotope fractionation by Fe-oxides, ranging from −16 to −27‰,^52^ but such leaching methods suffer from potential contamination by other secondary phases because no chemical reagent has absolute selectivity.

Most previous experimental studies of Li isotope fractionation during weathering have focused on Al-rich secondary minerals, such as gibbsite and phyllosilicates. Compared to these minerals, Fe-oxides such as goethite and hematite have no interlayers because Fe-oxide structures are usually close-packed.^53,54^ Gibbsite or clay minerals are therefore not suitable as analogues for understanding interactions of Li with Fe-oxides. In contrast to the suggestion that little Li is adsorbed by goethite mineral surfaces,^49^ experimental studies^55−57^ have used magic angle spinning nuclear magnetic resonance (MAS NMR) to demonstrate that Li can be adsorbed by goethite under pH conditions ranging from 4 to 11. The NMR characterization also suggests that the Li binding sites are different under different pH conditions.^55,57^ Given that goethite has a point of zero charge (PZC) value of 8.3 ± 0.9,^55,58^ it is intriguing that Li^+^ can be adsorbed onto goethite surfaces even in acidic environments where the surface should hold a positive charge.

The contrasting observations of Li behavior in Fe-rich geological materials and a lack of experimental work warrant new studies investigating the interaction between Li and Fe-rich minerals such as oxides. Here, we focus on the interaction between dissolved Li^+^ in aqueous solutions and a range of Fe-oxide minerals (goethite, hematite, wüstite, and magnetite) at pH values between 2 and 12. Through sorption experiments, we address how much Li is taken up by these Fe-oxides across a wide range of initial pH, assess the uptake mechanisms, and determine the Li isotope fractionation associated with sorption.

## 2 Materials and Methods

### 2.1 Materials

Six samples, including four different Fe-oxides, were employed in this study. Two goethite (FeOOH) and two hematite (Fe_2_O_3_) samples were used to represent fully oxidized Fe-oxides, whereas the mixed valence and less oxidized Fe-oxides were represented by magnetite (Fe_3_O_4_) and wüstite (FeO). The Fe-oxides were either synthesized (two goethite and one hematite sample) or commercially available (magnetite, wüstite, and one hematite sample). The Fe_3_O_4_ powder used as magnetite was iron (II, III) oxide (Aldrich 99.99%, Lot# MKBP9789 V). Iron(II) oxide was used as wüstite (Aldrich 99.9%, Lot# STBF3726 V). Iron(III) oxide powder (Aldrich ≥ 99%, Lot# MKBS6874 V) was used as a hematite sample. The samples synthesized on site (two goethite and one hematite sample) were produced in the laboratory following methods described by Cornell and Schwertmann.^53^ Hematite was prepared at high temperature by heating a 0.002 M HCl solution containing 0.02 M FeCl_3_ for 10 days at 98 °C. Goethite samples were synthesized at both low and high temperatures. Low-temperature goethite synthesis was achieved by bubbling air through a mixture of 110 mL 1 M NaHCO_3_ and 1 L 0.05 M FeCl_2_·4H_2_O solutions for 48 h at room temperature (∼21 °C). High-temperature goethite was synthesized by adding 180 mL 5 M KOH solution into 100 mL 1 M Fe(NO_3_)_3_, diluting to 2 L, heating at 70 °C for 60 h, washing with double-deionized water, and finally drying at 50 °C. To distinguish the two hematite samples, hematite synthesized in our laboratory is referred to as hematite_syn_, and hematite from a commercial source is referred to as hematite_com_. To distinguish the goethite synthesized using different methods, the goethite produced at low temperature is referred to as goethite_LT_, and the goethite synthesized at high temperature is referred to as goethite_HT_. The synthesized Fe-oxides were washed repeatedly with double-deionized water and freeze-dried. The specific surface area (SSA) of the oxide powders, as determined by nitrogen adsorption using the Brunauer–Emmet–Teller (BET) method at Utrecht University (UU), ranged widely from 0.137 to 146 m^2^/g (Table 1).

**Table 1:** Specific Surface Areas (SSA) of Fe-Oxides

oxide sample	description	SSA (m^2^/g)
goethite_LT_	synthesized at ∼21 °C	145.824 ± 1.196
goethite_HT_	synthesized at 70 °C	28.292 ± 0.198
hematite_syn_	synthesized at 98 °C	15.547 ± 0.198
hematite_com_	Aldrich	3.110 ± 0.233
magnetite	Aldrich	7.265 ± 0.039
wüstite	Aldrich	0.137 ± 0.059

### 2.2 Experiments

Two sets of sorption experiments were performed in the Geolab at UU. The first set of experiments (Experiment 1) investigated the effect of pH on the interaction between dissolved Li and various Fe-oxide particles. The second set of experiments (Experiment 2) studied Li uptake by Fe-oxide particles (goethite_LT_) as a function of time. In all of the experiments, a 0.1 M NaCl solution was used as the fluid matrix to minimize potential complex reactions between Fe-oxide particles and other dissolved ions.

In the Experiment 1 series, stock solution was prepared by diluting concentrated LiCl solution, which is made by dissolving LiCl (Carl Roth > 99%, Lot#212309558) in double-deionized water, using 0.1 M NaCl to obtain a Li concentration of ∼175 μM. Then, five substock solutions were prepared by adjusting the pH of each solution using either 0.1 M HCl or 0.1 M NaOH to reach the desired pH values of 1.98, 4.01, 5.96, 8.01, and 11.98. Before the experiment, the Fe-oxide particles (Table 1) were first preconditioned with a 0.1 M NaCl solution, recollected through centrifugation, and freeze-dried. Then, 10 mL of substock solution was added to approximately 0.2 g of Fe-oxide particles in 15 mL polypropylene centrifuge tubes, except for the hematite_com_ experiments in which less than 0.1 g of particles were used. In the subexperiments that used commercially obtained oxides, trace amounts of Li at the level of μg/g may have been present as impurities. However, their impact on the experiment is considered insignificant due to the high Li background concentration (∼175 μM) of the initial solution. The interaction experiments between fluid and Fe-oxide lasted for 30 days, with the suspension manually shaken twice a week and left at room temperature. Then, the samples were centrifuged at 4000 rpm to separate the aqueous solution from the solid Fe-oxides. An aliquot of the sample solutions (∼2 mL) was collected and filtered with 0.2 μm pore-size syringe filters for Li isotope and chemical analyses, and the remaining solution volumes were used for pH measurements.

In the Experiment 2 series, 2.494 g of goethite_LT_ particles were allowed to interact with 80 mL of mixed LiCl-NaCl solution. The solution initially had a pH of 12.03, a LiCl concentration of 36 μM, and a NaCl concentration of 0.1 M. The reaction was performed in a precleaned 100 mL borosilicate bottle, which was stirred with a magnetic stir bar at a room temperature of 21 ± 1 °C. The well-mixed solution was sampled after 1, 2, 4, 8, 16, 32, and 70 days of interaction. At each sampling point, 2 mL of sample mixtures containing both the reacting fluid and solids were pipetted and filtered using a 0.2 μm syringe filter.

Finally, a series of desorption experiments (Experiment 3) were conducted. At the end of Experiment 1, the Fe-oxide particles were carefully rinsed with double-distilled water and ethanol, filtered at 0.2 μm, and freeze-dried. Selected samples were allowed to react with extraction agents to investigate the desorption capacity of adsorbed Li. Two different agents were used to examine the effect of the pH on the extraction. For one experiment, ∼0.01 g reacted Fe-oxide particles were extracted using 2 mL of 1 M NH_4_Cl solution at a pH of 4.84. In a separate experiment, ∼0.05 g of Fe-oxide particles were extracted using 3 mL of 1 M NH_4_OAc at a pH of 7.26. The extraction experiments were conducted in 15 mL polypropylene centrifuge tubes, and the samples were shaken for 24 h, with the extracted solutions collected by centrifugation at 4000 rpm and filtration at 0.2 μm. In all of our experiments, no unforeseen or unusually high safety risks were identified.

### 2.3 Fluid Chemical and Isotopic Analyses

Measurements of Li concentrations were conducted in the Geolab at UU for high-concentration samples ([Li] > 144 μM or 1 μg/mL) and at Institute de Physique du Globe de Paris (France) for samples with lower Li concentrations ([Li] < 144 μM or 1 μg/mL). All samples were redissolved in 0.7 M HNO_3_. High-concentration samples were measured by inductively coupled plasma mass spectrometry (ICP-MS, NeXION 2000P) and were calibrated using a set of standards with concentrations ranging from 0 to 10.8 μM (or 75 ng/mL). The detection limit ranged from 0.14–1.44 μM (or 1–10 ng/mL), depending on operational conditions. Low-concentration samples were measured by inductively coupled plasma quadrupole mass spectrometry (ICP-Q-MS, Agilent 7900) and were calibrated using a set of standards with concentrations ranging from 0.14 to 28.81 μM (or 1–200 ng/mL). Independent standard solutions with concentrations of 10–100 ng/mL were prepared in-house by diluting certified quality control standards (QCP-QCS-1 and IV-28, Inorganic Ventures) and measured to determine analytical accuracy. Analytical uncertainties for measurements at both laboratories were below 10%. Several samples were measured in both laboratories and had concentration differences that were within 2%. Sodium contents were measured by inductively coupled plasma optical emission spectroscopy (ICP-OES, PerkinElmer Avio 500) in the Geolab at UU with an analytical uncertainty better than 10%.

A double-step separation protocol using AG50W X-12 200–400 mesh cation exchange resin and elution with 0.2 M HCl^11^ was followed to purify Li from the sample matrix prior to Li isotope measurements of the aqueous solutions.^46,52,59^ The Li isotope composition of the purified samples was measured at the LOGIC laboratories at University College London (United Kingdom) using a Nu Plasma 3 multi-collector inductively coupled plasma mass spectrometer (MC-ICP-MS) coupled to a CETAC Aridus III desolvating nebulizer system. An IRMM-016 solution was used as the bracketing standard to correct for instrumental mass fractionation. Atlantic seawater and blanks were processed together with the experimental samples to check the quality of the Li purification and Li isotope measurement. The IRMM-016 standard had an intensity of ∼17 pA for a 1 ng/mL solution (∼1.7 V/ppb), the background solution (2% HNO_3_ v/v) had an intensity less than 0.02 pA, and the total procedural blank had a signal of 0.09 pA, registering a negligible effect (<0.2% of total Li) on the Li isotope measurements. Two Atlantic seawater samples were measured, with δ^7^Li values (30.9 ± 0.5 and 30.7 ± 0.1‰) in good agreement with previously reported seawater values.^11,60^ Measurement uncertainties are, in general, better than 0.5‰ (2 s.d), and the long-term external error, based on seawater analyzed over a period of several years, is ±0.4‰ (2 s.d., *n* = 52).^46^

### 2.4 Characterization of Fe-Oxide Particles

All of the Fe-oxide particles were characterized in the Geolab at UU using a Bruker-AXS D8 ADVANCE X-ray diffractometer (XRD) DAVINCI design with a LYNXEYE XE-T detector (with 192 measuring points) and a θ/θ goniometer. The accuracy was 0.01° 2θ. In brief, ∼1 g of the bulk sample was loaded and scanned from 3 to 80 2θ (°) using Cu Kα X-ray radiation, and ∼0.1 g of samples recovered from the experiment was scanned from 5 to 80 2θ (°). Solid samples were also characterized by attenuated total reflectance–Fourier transform infrared spectroscopy (ATR-FTIR) and Raman spectroscopy. Raman spectra were acquired on a WITEC Alpha 300 system equipped with a 532 nm laser and a grating of 600 grooves/mm. Spectra were acquired for 30 seconds to provide sufficient signal to noise ratios. The ATR-FTIR measurements were performed using a Thermo Fisher Scientific Nicolet 6700 instrument equipped with a GladiATR monolithic diamond crystal ATR accessory. Selected goethite samples were analyzed at the Electron Microscope Centre at UU using a Zeiss Gemini 450 scanning electron microscope (SEM) and a Thermo Fisher Talos F200X (scanning) transmission electron microscope ((S)TEM) to examine the main morphological features and nanostructures of the goethite particles.

## 3 Results

**Figure 1:**
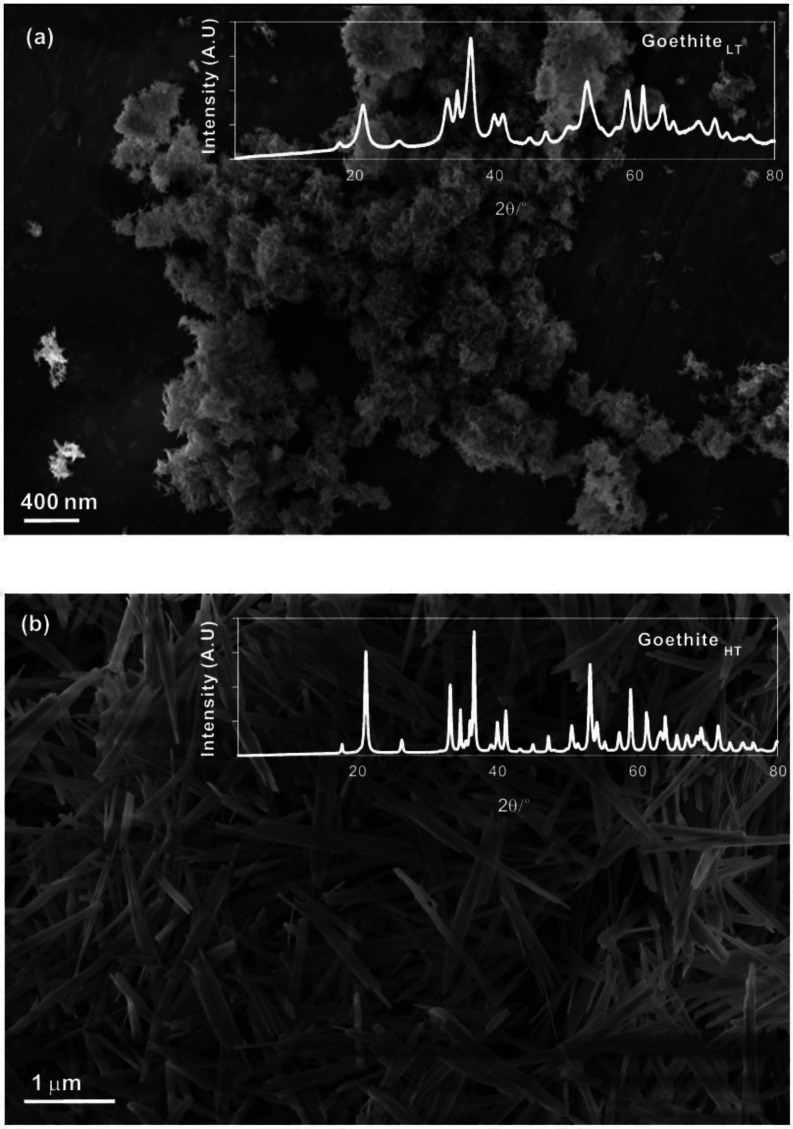
SEM characterization of (a) goethite_LT_ and (b) goethite_HT_ particles. Inset graphs show XRD results (Figure S1).

**Figure 2:**
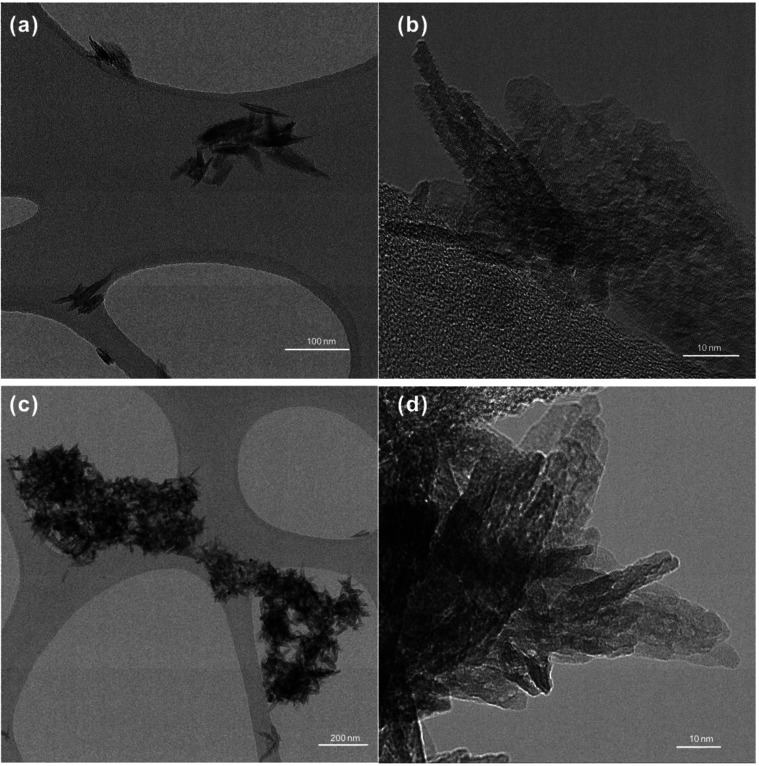
Solid characterization of goethite_LT_ particles using transmission electron microscopy (TEM) and high-resolution TEM (HRTEM): (a) unreacted goethite_LT_ particles; (b) particles from (a) observed under HRTEM; (c) goethite_LT_ particles interacted with a solution of pH ∼ 12; and (d) particles from panel (c) observed under HRTEM.

The XRD patterns and ATR-FTIR absorbances of the Fe-oxides used for this study are displayed in Figures S1 and S2. Notably, goethite_HT_ exhibits a higher crystallinity than goethite_LT_, as indicated by the smaller width at half-height of the XRD and ATR-FTIR bands for goethite_HT_. Imaging by SEM further demonstrates the differences between goethite_LT_ and goethite_HT_ (Figure 1a,b). The goethite_HT_ grains display a well-defined mineral morphology, with clear facets and smooth mineral surfaces (Figure 1b). The grain lengths were >1 μm, and widths generally ranged from ∼100 to ∼200 nm. Less defined surficial features were observed in the goethite_LT_ particles (Figure 1a). These particles are significantly smaller than those synthesized at high temperatures (goethite_HT_ particles), and their characteristics can only be observed under TEM, which has a higher spatial resolution. The goethite_LT_ particles had grain lengths varying from ∼50 to ∼70 nm and widths ranging from 5 to 10 nm (Figure 2a,b).

**Figure 3:**
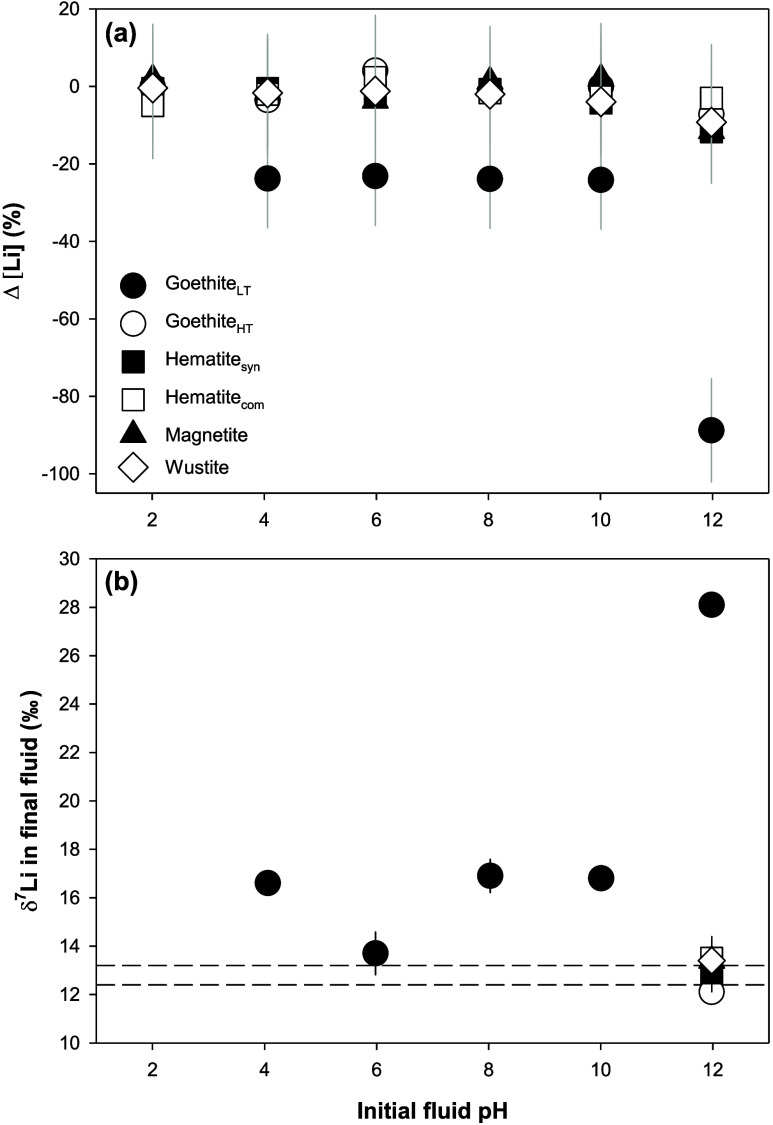
Lithium sorption onto Fe-oxides at various initial pH values: (a) changes of fluid Li content in percentage under different initial pH conditions with various Fe-oxides from Experiment 1; and (b) δ^7^Li signatures in fluids at the end of sorption from selected samples, with the initial δ^7^Li signature of the LiCl stock solution marked by the dashed lines (12.8 ± 0.4‰).

In the Experiment 1 series, with the exception of the goethite_LT_ experiments, no significant Li uptake by Fe-oxides was observed across the pH_i_ (i denotes initial) range from 2 to 10 (Figure 3). Although an ∼10% decrease in fluid Li content was observed for the experiments at pH_i_ ∼ 12, this difference is within the margin of uncertainty and therefore not significant. In the experiments where goethite_LT_ was the sorbing substrate, ∼25% Li was removed from the fluid phase when pH_i_ was between 4 and 10, and ∼90% Li was removed at pH_i_ ∼ 12 (Figure 3 and Table 2).

**Table 2:** Experiment 1: Li Sorption by Fe-Oxide Powders (Goethite, Hematite, Magnetite, and Wüstite) at Various pH Conditions

sample	mass of oxides (g)	pH_i_	pH_f_	[Li]_i_ (μmol/L)^a^	[Li]_f_ (μmol/L)^a^	[Na]_i_ (mmol/L)^a^	[Na]_f_ (mmol/L)^a^	expected initial surface charge based on PZC^b^	final δ^7^Li in solution	2 s.d.
goethite_LT_ synthesized at 21 °C										
GX2	0.1989	2.01	4.15	180.70	180.07	108.01	115.31			
GX4	0.1924	4.06	7.68	185.55	141.41	107.02	110.47	+	16.6	0.4
GX6	0.1969	5.98	7.68	181.38	139.35	108.85	110.50	+	13.7	0.9
GX8	0.1973	8.03	7.67	183.03	139.25	108.35	111.73	–	16.9	0.7
GX10	0.1914	10.01	7.64	185.94	141.06	106.43	112.36	–	16.8	0.3
GX12	0.1943	11.98	9.72	188.18	21.12	117.46	116.82		28.1	0.5
goethite_HT_ synthesized at 70 °C										
GN2	0.2071	2.01	2.09	180.70	180.63	108.01	108.10			
GN4	0.2050	4.06	6.73	185.55	178.96	107.02	109.28	+		
GN6	0.2034	5.98	6.92	181.38	188.75	108.85	110.80	+		
GN8	0.2048	8.03	6.99	183.03	180.38	108.35	110.43	–		
GN10	0.2070	10.01	7.21	185.94	185.80	106.43	110.97	–		
GN12	0.1995	11.98	11.91	188.18	174.68	117.46	119.47		12.1	0.3
synthesized hematite_syn_										
HX2	0.2077	2.01	1.96	180.70	179.68	108.01	104.77			
HX4	0.2252	4.06	3.78	185.55	184.52	107.02	110.74	–		
HX6	0.1995	5.98	4.06	181.38	178.59	108.85	108.39	–		
HX8	0.2004	8.03	4.21	183.03	181.64	108.35	108.69	–		
HX10	0.2005	10.01	4.14	185.94	177.99	106.43	105.65	–		
HX12	0.2013	11.98	11.85	188.18	166.12	117.46	114.99		12.9	0.8
commercially available hematite_com_										
HN2	0.0378	2.01	1.97	180.70	171.73	108.01	110.23			
HN4	0.0472	4.06	4.33	185.55	181.75	107.02	107.47			
HN6	0.0991	5.98	6.07	181.38	185.55	108.85	107.53	+		
HN8	0.0896	8.03	6.32	183.03	179.98	108.35	109.48	–		
HN10	0.0334	10.01	6.96	185.94	183.76	106.43	107.19	–		
HN12	0.0493	11.98	12.03	188.18	182.51	117.46	115.50			
commercially available magnetite										
M2	0.1920	2.01	2.13	180.70	184.15	108.01	106.30			
M4	0.1012	4.06	6.85	185.55	182.84	107.02	107.12	+		
M6	0.1993	5.98	7.03	181.38	174.80	108.85	112.07	+		
M8	0.1981	8.03	7.06	183.03	185.44	108.35	114.24	–		
M10	0.2166	10.01	7.47	185.94	189.69	106.43	110.81	–		
M12	0.1977	11.98	11.95	188.18	166.59	117.46	117.74		13.5	0.6
commercially available wüstite										
W2	0.1988	2.01	3.91	180.70	179.94	108.01	110.27			
W4	0.2270	4.06	10.08	185.55	182.42	107.02	103.57	+		
W6	0.1992	5.98	10.26	181.38	179.17	108.85	107.39	+		
W8	0.1729	8.03	10.14	183.03	179.38	108.35	108.66	+		
W10	0.1752	10.01	10.15	185.94	178.53	106.43	109.03	+		
W12	0.1876	11.98	11.92	188.18	170.80	117.46	122.40		13.4	1.0

a Analytical uncertainty is ±10%.

b PZC is reflected in the pH_f_ when this is consistent within 1 pH unit and across several initial pH conditions. Under these conditions, it is expected that charge neutrality is achieved via interaction with solution ions only; therefore, the expected initial charge is not given for the highest and lowest pH experiments, which deviate in their final pH.

**Figure 4:**
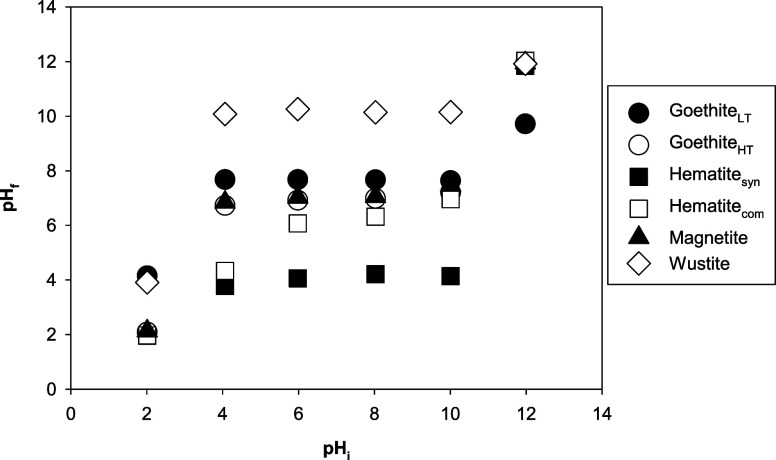
Variations in fluid pH at the beginning (pH_i_) and the end (pH_f_) of the Li-sorption experiments.

A “buffer” effect was observed in the experiments based on the difference between pH_i_ and pH_f_ (f denotes final) when the pH_i_ was between 4 and 10. In these cases, pH_f_ reached very similar values when the same phase was used despite the different initial pH values (Figure 4): 7.67 ± 0.02 for goethite_LT_ experiments, 6.96 ± 0.17 for goethite_HT_, 4.05 ± 0.16 for hematite_syn_, 5.92 ± 0.97 for hematite_com_, 7.10 ± 0.23 for magnetite, and 10.15 ± 0.06 for wüstite. The experiments conducted at pH_i_ of ∼2 and ∼12 did not follow this trend and instead remained at a similar pH throughout the experiment, except for the experiments with goethite_LT_ where the pH was shifted by ∼2 units toward neutral in both experiments. A similar shift was observed for wüstite in the experiment at pH_i_ ∼ 2 but not at pH_i_ ∼ 12.

As most of the experiments showed a similar behavior in their pH_f_ and minimal to no Li uptake, only selected samples were analyzed for their δ^7^Li signatures, including samples from the goethite_LT_ experiments and samples from the most alkaline experiments for all Fe-oxide types (pH_i_ ∼ 12). For the goethite_LT_ experiments, all of the solutions were either slightly or significantly enriched in the heavy Li isotope ^7^Li compared to their initial LiCl-NaCl solution, which had a δ^7^Li value of 12.8 ± 0.4‰ (Figure 3b). The solutions from the goethite_LT_ experiments conducted at pH ranging from 4 to 10 resulted in a similar pH_f_ (∼7.6) and degree of Li sorption (∼25%) and also had relatively similar δ^7^Li signatures (16.0 ± 1.3‰). The experiment with the highest uptake at pH_i_ ∼ 12 had the highest δ^7^Li value of 28.1 ± 0.5‰. In contrast, the experiments conducted at pH_i_ ∼ 12 with the other Fe-oxides all produced δ^7^Li values that were within the error of their initial LiCl-NaCl solution value (Figure 3b).

**Figure 5:**
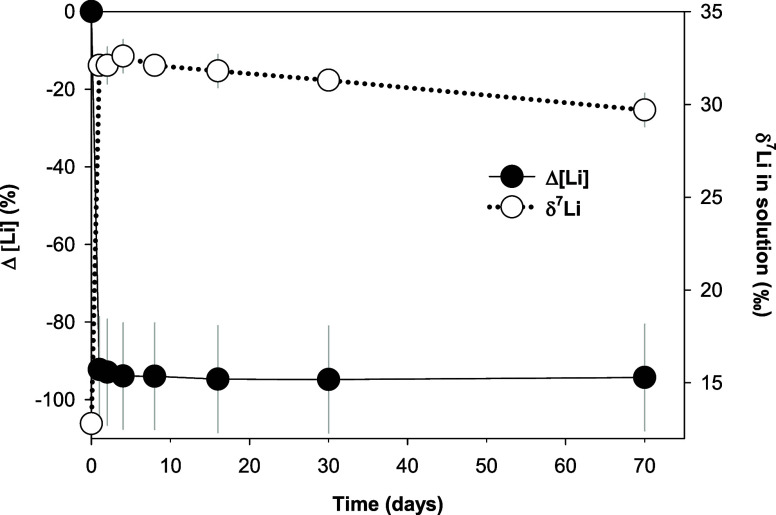
Lithium sorption and isotope fractionation by goethite_LT_ at pH_i_ ∼ 12 from Experiment 2. Changes in fluid Li content are represented in %.

In the Experiment 2 series, fluid Li was rapidly taken up by goethite_LT_, with the dissolved Li content decreasing from ∼36 to ∼3 μM within 1 day, after which the concentration of Li in solution remained stable (Table 3, Figure 5). The fluid samples from Experiment 2 were also analyzed for their δ^7^Li signatures, revealing a consistent enrichment of ^7^Li in solution during fluid interaction with goethite_LT_. Compared to the initial LiCl-NaCl solution (δ^7^Li = 12.8‰ ± 0.4), the final solutions of the goethite_LT_ subexperiments had δ^7^Li signatures that varied from 29.7 to 32.6‰. This change in the Li isotope composition directly corresponds to the rapid removal of Li from the solution and the change in the pH within the first day of this experiment (Table 3).

**Table 3:** Experiment 2: Li Sorption through Time by Goethite_LT_ Powders with a Starting pH of 12

sample	elapsed time (days)	[Li] (μmol/kg)^a^	δ^7^Li in solution	2 s.d.	pH	[Na] (mmol/kg)^a^
LiCl-NaCl solution		36.2	12.8	0.4	12.03	116.22
D1	1	2.8	32.1	0.4	9.73	113.56
D2	2	2.6	32.1	1.0		111.54
D4	4	2.2	32.6	0.9		115.61
D8	8	2.2	32.1	0.6	9.68	113.03
D16	16	1.9	31.8	0.9		116.95
D30	30	1.9	31.3	0.2		115.29
D70	70	2.1	29.7	0.9	9.59	114.63

a Analytical uncertainty is ±10%.

Selected samples of solid goethite_LT_ recovered from Experiments 1 and 2 were characterized by TEM, ATR-FTIR, XRD, and Raman spectroscopy. No significant difference was observed in the mineral morphology between the reacted and unreacted goethite_LT_ particles (e.g., Figure 2c,d cf. Figure 2a,b). Shifts of ATR-FTIR absorbance were observed between the unreacted goethite_LT_ particles and the goethite_LT_ particles recovered from the sorption experiments. At ca. 630 cm^–1^, the band positions of reacted goethite_LT_ particles from Experiments 1 and 2 are shifted to lower wavenumbers compared to those of unreacted goethite_LT_ particles (Figure S3). For comparison, the peak positions of unreacted goethite_HT_ particles were also analyzed, and they showed the same bands, but the band close to 630 cm^–1^ was found to occur at a higher wavenumber than determined for the goethite_LT_ particles (Figure S3). Minor differences were observed in the XRD patterns (Figure S4) and Raman spectra (Figure S5) between the reacted and unreacted goethite_LT_ powders.

In the Experiment 3 series, Li taken up by goethite_LT_ during the Experiment 1 series was extracted from the reacted goethite_LT_ particles. When NH_4_OAc was used to extract the Li, less than 3% was released back into solution, whereas significant amounts (50–82%) of Li were liberated when NH_4_Cl was used as the extracting agent (Table 4).

**Table 4:** Experiment 3: Li Desorption by Extracting with NH_4_Cl and NH_4_OAc

sample	mass (g)	Li adsorbed from Exp 1 (ng)^a^	[Li] in extraction solution (μg/kg)	fraction extracted (%)
Li extraction with 2 mL NH_4_Cl (pH = 4.84)				
GX4	0.0090	143.33 ± 75.68	59.08 ± 5.91	82.44 ± 44.30
GX12	0.0075	447.59 ± 50.71	113.10 ± 11.31	50.54 ± 7.64
Li extraction with 3 mL NH_4_OAc (pH = 7.26)				
GX4	0.0595	916.60 ± 493.96	4.20 ± 0.42	1.37 ± 0.75
GX12	0.0490	2924.26 ± 331.46	24.99 ± 2.50	2.56 ± 0.37

a Li adsorbed from Experiment 1 is calculated as ([Li]_i_ – [Li]_f_) × 10 mL × 6.941 g/mol × sample mass (used in Experiment 3)/sample mass (used in Experiment 1); [Li]_i_, [Li]_f_, and mass used in Experiment 1 are from Table 2.

## 4 Discussion

### 4.1 Lithium Sorption onto Fe-(Oxyhydr)oxides

In all of the experiments conducted between pH values of 4 and 10, there is a buffering effect of the Fe-oxide on the pH of the solution (Figure 4). This feature has been described previously in the literature for Fe-oxides, including goethite,^61^ hematite,^62^ and magnetite.^63^ The attainment of a consistent pH_f_ across a range of pH_i_ values reflects the electrostatic interaction of negatively or positively charged ions within the solution at the sample surface to achieve charge neutrality. In previous experiments, the pH_f_ value has been demonstrated to reflect the point of zero charge for a material under the chemical conditions of the solution.^61−63^ Therefore, we expect that our systems have attained charge neutrality by the end of the experiments. This scenario means that there is no overall attractive force expected to occur in the experiments between the surface of the mineral and the ions in the fluid at equilibrium.

However, when the pH_i_ was above the pH_f_, and hence the surface was negatively charged (Table 2), the uptake of positively charged ions, such as Li^+^ or Na^+^, at the mineral surface could be expected to have occurred during the equilibration process.^61,63^ Based on the changing pH observed in Experiment 2 (Table 3) and previous studies,^61,63^ such a process can be expected to have occurred quickly, within the first 24 h of the experiments. However, no changes in the Na^+^ or Li^+^ concentrations in solution that would reflect attractive forces based on the expected mineral surface charge and PZC were observed with any of the Fe-oxides, except for goethite_LT_ (Table 2). The goethite_LT_ samples showed an overall Li uptake over the entire pH range studied (Figure 3a), where the uptake does not correlate with the expected cation exclusion effects in the experiments conducted at pH_i_ values of ∼4 and ∼6, which should have a positively charged surface based on the PZC of this sample at pH 7.67. This finding is consistent with previous experiments, which have shown that positively charged ions only very weakly interact with negatively charged Fe-oxide surfaces in the form of an outer-sphere complex,^64^ and hence we conclude that the observed Li uptake by goethite_LT_ is not driven by outer-sphere electrostatic adsorption.

A lack of inner-sphere adsorption complexes has previously been demonstrated for Li^+^ on magnetite^63^ and hematite^65^ using potentiometric methods, even at solution Li concentrations above those expected in the natural environment or used here. However, this lack of direct interaction between Li^+^ and Fe-oxide surfaces is contradicted by more recent NMR studies focusing on goethite nanoparticles.^55,57^ Here, evidence for direct interactions involving Fe–O–Li (inner-sphere complexation) on nanoparticulate goethite synthesized at room temperature was observed after sample drying at pH values above the measured PZC. Direct interaction between the solid phase and Li^+^ in solution was also present in our experiments with goethite_LT_. In contrast to the study of Nielsen et al.,^55^ our goethite_LT_ experiments demonstrated Li loss from the solution across the entire pH range. This finding corresponds with an increase in the PZC of 0.71 pH units from that of goethite_HT_ to that of goethite_LT_ (Figure 4). Given that no evidence for an additional phase was observed in SEM, TEM, or XRD analyses, the Li uptake by the solid and the change in the PZC imply that a chemical change may have occurred to the goethite surface during the experiments. This chemical change to the goethite_LT_ is supported by the ATR-FTIR absorbance shift (Figure S3) at peak positions that correspond to the symmetric Fe–O stretching band (ca. 630 cm^–1^).^66^ Decreases in goethite crystallinities result in a shift toward lower wavenumbers of this band.^66^ Among the analyzed goethite samples, the frequencies of this band decrease in an order from goethite_HT_ particles with the highest frequencies (634.2 cm^–1^) to unreacted goethite_LT_ particles (629.5 cm^–1^) and finally to reacted goethite_LT_ particles with the lowest frequencies (∼610–618 cm^–1^). This finding suggests that chemical changes of the goethite_LT_ particles occurred during the sorption experiments. At mineral surfaces, a typical cation uptake reaction can involve dissolution and reprecipitation, which is driven by the neoformation of the solid phase.^67^ Hence, our observations could be explained by the reprecipitation at active sites on the poorly crystalline goethite_LT_ surface. Unfortunately, it is not clear from the Nielsen et al. study^55^ whether any pH changes were observed during their experiments. Therefore, we cannot presently evaluate whether the minerals in their system behaved in a similar manner, but their observations of apparent inner-sphere complexes at the surface could potentially reflect the neoformation of a solid phase, with Li occupying sites other than the OH-site within the goethite channels.

The extraction test in Experiment 3 demonstrated that only minimal Li^+^ could be extracted from the goethite_LT_ at near-neutral pH values, whereas there was significant extraction in an acidic environment (Table 4). A mineral phase is expected to have minimal solubility close to its PZC,^63^ so the goethite_LT_ sample is expected to have a minimal solubility at pH values close to 7.67. The restricted extraction of Li from the samples using NH_4_OAc reflects this feature, as this solution has a pH of 7.26, and only a very small fraction of adsorbed Li was released (∼1.4% for goethite_LT_ reacted at pH ∼ 4, and ∼2.4% for goethite_LT_ reacted at pH ∼ 12). In contrast, during the extraction in an acidic environment with NH_4_Cl (pH of 4.84), a significant portion of the originally adsorbed Li was extracted, with release of 82.4% of the Li adsorbed on goethite_LT_ reacted at pH ∼ 4, and 50.5% of the Li adsorbed on goethite_LT_ reacted at pH ∼ 12. We note that during the goethite_LT_ particle recovery through rinsing and filtration, some adsorbed Li may have been removed by rinsing with water.^28^ Therefore, these results may provide only a lower limit on the extraction capacity.

### 4.2 Lithium Sorption onto Poorly Crystalline Goethite_LT_ Particles and Associated Li Isotope Fractionation

In the Experiment 1 series, the fluid pH_f_ values imply different systematic behavior under the tested pH range, as discussed in Section 4.1. In general, pH_f_ tends to deviate from pH_i_ to reach the PZC when pH_i_ ranges from 4 to 10, whereas at pH_i_ of 2 or 12, the reacted solutions have pH_f_ values close to pH_i_. Therefore, the Fe-oxides likely underwent different reactions, such as dissolution at pH ∼ 2 and possible reprecipitation at pH ∼ 12. This variation could also have resulted in different interactions between fluid Li and reacted Fe-oxides. Lithium uptake was only observed with goethite_LT_, and indeed, this Li uptake was controlled by pH_i_: at pH ∼ 2, the system likely prefers goethite_LT_ dissolution, and no Li uptake was observed, whereas at higher pH_i_ values from 4 to 12, Li uptake became significant. Furthermore, the Li uptake capacity of goethite_LT_ varied with pH, with only ∼25% Li adsorbed for pH_i_ ranges from 4 to 10, increasing to ∼90% uptake of Li at pH_i_ ∼ 12 in both Experiments 1 and 2, whereas the changes in solution Na content were minor (Tables 2 and 3). Hence, the mechanisms driving the Li uptake may have varied, as suggested by differences in pH_f_ (Figure 4) and by previous NMR studies.^55,57^

We suggest that the uptake of Li by goethite_LT_ can be attributed to Li incorporation on poorly crystalline goethite_LT_ surfaces through dissolution and reprecipitation at active sites and that two different neoformations of solid phases, for instance, two different materials, may occur at pH_i_ from 4 to 10 and at pH_i_ ∼ 12. Our Li sorption results are in agreement with previously reported Li adsorption behavior traced by ^6^Li MAS NMR spectra.^55^ That study showed an elevated Li adsorption capacity of goethite with increasing pH and suggested that adsorbed Li can be located in different inner-sphere sites. Interestingly, in the NMR characterization, a ^6^Li peak was detected in their goethite particles (with particle size smaller than goethite_LT_ used in the current study) when reacted with dissolved Li at pH_i_ ∼ 4, which is much lower than the PZC of goethite. Nielsen et al.^55^ suggested that the presence of Li in the goethite particles could be due to (i) a pH change during the experiment or (ii) Li precipitation during the goethite recovery at the end of the adsorption experiment (isolation and drying).^55^ At pH > PZC, NMR results suggest that Li can be bound to a bidentate edge site associated with two FeOH groups, or at high pH a pocket site associated with a deprotonated Fe_3_OH group and FeOH group.^57^

Because we monitored the changes of Li concentration in the fluid, our experimental data demonstrate that Li sorption indeed takes place when the initial solution pH is significantly lower than the PZC (e.g., pH ∼ 4). In addition, the reacting fluids showed an increase in pH from 4.06 to 7.68 at the end of the experiment. If the Li uptake was driven by electrostatic forces, positively charged Li cations would not be taken up under pH conditions lower than those of the PZC. Therefore, our observations support the assumption that Li uptake is caused by a fluid-goethite reaction via neoformation.

We also note that the solubility of goethite varies with pH, with higher solubilities at both acid (pH < 6) and alkaline (pH > 10) conditions.^68−70^ Furthermore, we note that the goethite_LT_ grain surfaces are not well defined, which is indicated by their roughness (Figure 2). A possible mechanism during fluid-goethite_LT_ interactions could be provoked by the partial dissolution of FeOOH at defect-containing goethite surfaces, thus containing active sites.^71^ Various aqueous Fe species could be formed, such as Fe(OH)_2_^+^ in acidic pH or Fe(OH)_4_^–^ at alkaline conditions.^68−70^ The reprecipitation or readsorption of this temporarily dissolved Fe back onto the goethite surface could essentially form new molecules, which take up cations such as Li from the ambient solution. A first-order observation can be made from our results that the Li sorption capacity is related to the SSA (Tables 1 and 2), which can be explained by the higher population of active sites in poorly crystalline particles, which in turn would result in both a larger SSA and greater potential for reprecipitation reactions.

Geochemical modeling using PHREEQC^72^ suggests the potential formation of hematite throughout the pH range used in Experiment 1, and fluid chemistry modeling using HSC Chemistry software (version 9) suggests the possible presence of LiFe_5_O_8_ under alkaline conditions (Figure S6). We note that the modeled results may not be fully indicative because the actual solubility of goethite_LT_ surficial materials is unknown, and the precipitated phases are likely amorphous and, therefore, not available in the PHREEQC database. For the fluid chemistry modeling, we have opted to use a fluid system with relatively high Fe and Li contents to maximize the potential formation of Li-carrying Fe-oxides. In spite of the limitations, the modeled results support the formation of a new oxide phase incorporating Li and Fe preferentially under alkaline conditions, as suggested by Experiments 1 and 2. Our extraction results (Experiment 3) can therefore be explained by a higher solubility of this neoformed solid phase in an acidic environment.

In the experiments with goethite_LT_, the Li uptake was accompanied by Li isotope fractionation, with light ^6^Li preferentially taken up by the solid phase. The Li isotope fractionation in the fluid system follows the isotope mass balance
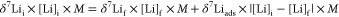
1where *M* is the fluid mass and δ^7^Li_ads_ is the Li isotope signature of the adsorbed Li on the goethite_LT_ particles, which can be calculated because values for all of the other terms in eq 1 are available in Tables 2 and 3. Direct measurements of δ^7^Li_ads_ values are not possible due to the challenge associated with isolating the Li taken up by goethite nanoparticles from the Li in the reacting fluids. The Li isotope fractionation during Li uptake by Fe-oxides can then be calculated as
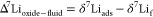
2and the associated Li isotope fractionation factor (α) can be determined based on the processes driving the fractionation. Here, there are two possibilities: either (i) equilibrium fractionation, if the neoformed Li-containing phase forming via surface reactions is in a continuous chemical equilibrium with the fluid, or (ii) Rayleigh fractionation, if the Li precipitated in the newly formed solid phase removes Li from the fluid via fractional distillation.

**Figure 6:**
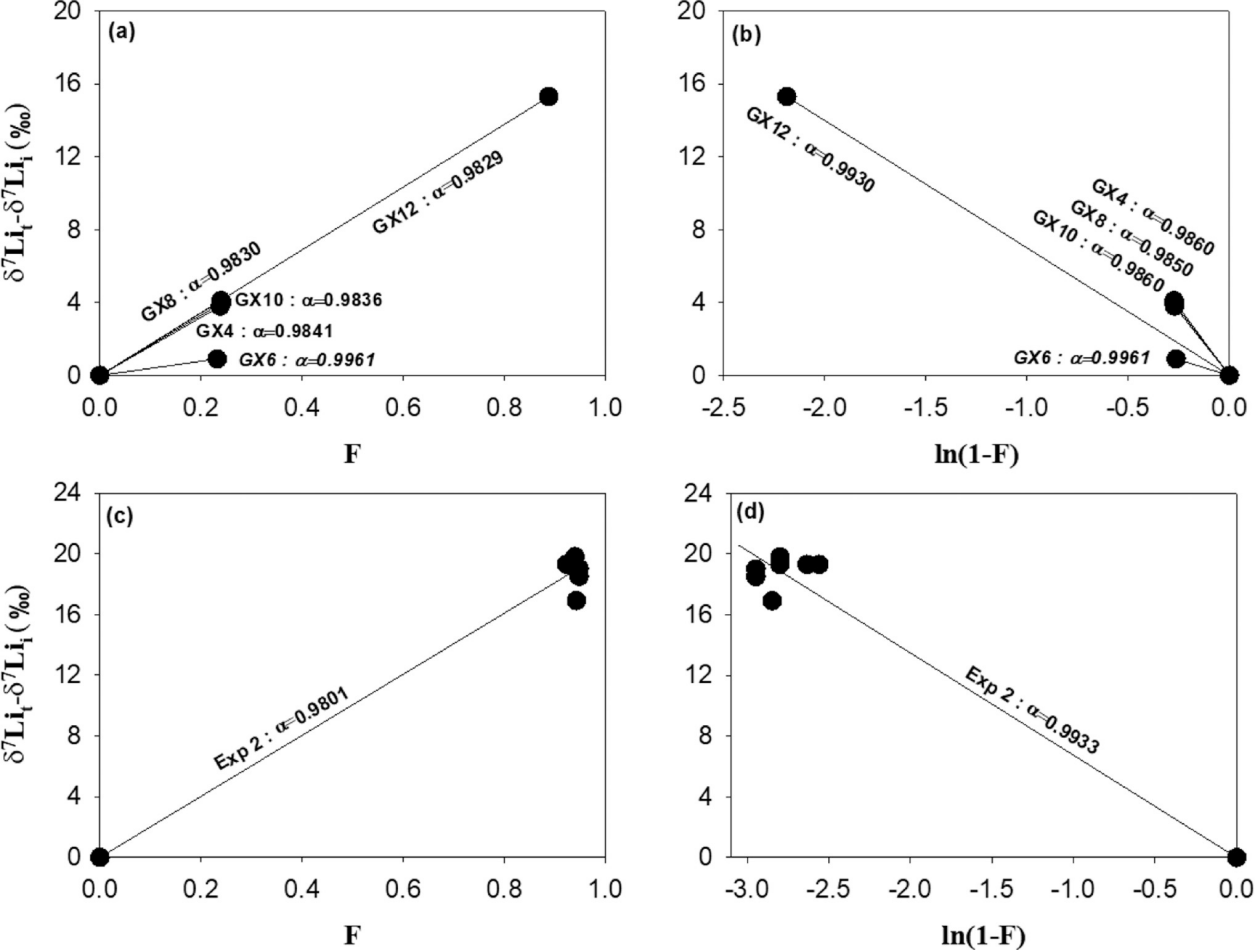
Estimation of Li isotope fractionation factors for (a) Experiment 1 in the scenario of equilibrium fractionation; (b) Experiment 1 in the scenario of Rayleigh fractionation; (c) Experiment 2 in the scenario of equilibrium fractionation; and (d) Experiment 2 in the scenario of Rayleigh fractionation.

Under circumstance (i), α can be calculated as
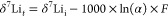
3where *t* denotes the time of sampling and *F* is the fraction of Li taken up by the solid phase, which is calculated as
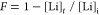
4On the other hand, under circumstance (ii), the α value can be estimated from
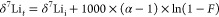
5In Experiment 1, although the Li uptake mechanisms by goethite_LT_ may differ between pH conditions (e.g., pH_i_ 4–10 vs pH_i_ ∼ 12), the calculated Δ^7^Li_oxide-fluid_ values of samples with different pH_i_ values indicate only minor deviations in the isotope fractionation (Table S1). The Li isotope fractionation associated with Li sorption by goethite_LT_ nanoparticles averages Δ^7^Li_oxide-fluid_ = −16.7 ± 0.5‰. We excluded a single data point from the experiment performed at a pH_i_ of 5.98 (Table 3), the Li isotope fractionation of which is insignificant. The reasons for this difference are unclear but may potentially be an analytical artifact, such as ineffective isolation of the reacting fluids from the goethite nanoparticles or poor instrumental performance for this sample. The associated fractionation factor in the scenario of equilibrium fractionation is α = 0.9834 ± 0.0005 (*n* = 4) (Figure 6a). In the scenario of Rayleigh fractionation, two α values were determined (Table S1): Li sorption by goethite_LT_ at pH values ranging from 4 to 10 has a similar fractionation factor of α = 0.9855 ± 0.0004 (*n* = 3), whereas at pH ∼ 12, the value becomes 0.9930 (Figure 6b).

In Experiment 2, the average fractionation and the fractionation factor were estimated between each sampling point and the initial solution, as the experiment quickly reached dynamic equilibrium in terms of Li concentration (Δ[Li] ∼ 0) in less than 1 day (Figure 5). A fractionation of Δ^7^Li_oxide-fluid_ = −20.1 ± 1.0‰ (*n* = 7) was observed (Table S1). In the case of equilibrium fractionation, the associated fractionation factor is α = 0.9801 (Figure 6c), which is slightly different from the α value (0.9829) calculated in Experiment 1 under the same pH conditions. In the case of Rayleigh fractionation, α = 0.9933 (Figure 6d), and this value is close to the one from Experiment 1 under the same scenario (α = 0.9930; Figure 6b).

A difference of ∼3‰ in Δ^7^Li_oxide-fluid_ (i.e., ∼−17‰ vs ∼−20‰) is observed between the results obtained from Experiments 1 and 2, which essentially leads to the small difference in the Li isotope fractionation factors calculated in the scenario of equilibrium fractionation. With the current data set, we are unable to determine the cause of this difference. However, there were some differences in the design of these two experiments, which could potentially account for such a difference: (1) in Experiment 1, the goethite_LT_ particles were equilibrated with 0.1 M NaCl before interacting with the mixed NaCl-LiCl solutions at various pH values, whereas in Experiment 2, the goethite_LT_ particles were not pretreated with a NaCl solution and were directly mixed with NaCl-LiCl at pH ∼ 12; (2) in Experiment 1, all of the samples were manually shaken with the Fe-oxide particles settled at the bottom of the centrifuge tube, whereas the sample in Experiment 2 was rigorously stirred, which ensured the sample mixture remained well mixed; and (3) different initial Li concentrations were used, with [Li]_i_ = 175 μM in Experiment 1 and [Li]_i_ = 36 μM in Experiment 2.

Because Na is typically considered mobile and is not taken up by secondary phases,^47,73,74^ as also observed in our experiments (Tables 2 and 3), the evolution of the Li/Na ratio in the fluid reflects the Li uptake by the oxides and varies accordingly with fluid δ^7^Li values. The coevolution of δ^7^Li values and Li/Na ratios in Experiment 2 is compared between the measured data (Table 3) and the modeled results calculated with eqs 3–5 for equilibrium fractionation and Rayleigh fractionation scenarios (Figure S7). The two scenarios remain unresolvable for Experiment 2 with the current data set.

In Experiment 1, under the scenario of equilibrium fractionation, similar fractionation factors are obtained from the subexperiments of goethite_LT_ at pH values of 4–12 (Figure 6a). This feature could be explained by reprecipitation through Ostwald ripening, which dissolves smaller particles, possibly at surface defects, and reprecipitates solid phases.^75^ In this case, Li uptake through reprecipitation during the interaction between fluid Li and goethite_LT_ has similar α values at different pH_i_ values (Figure 6a), even though the neoformed phase may be different, and the fractionation factors are comparable to those obtained from Li interactions with poorly crystalline kaolinite.^12^ On the other hand, in the scenario of Rayleigh fractionation, the two different α values determined with different pH_i_ values (4–10 vs 12) suggest two different isotope fractionation factors, which could be attributed to different solid chemistry during reprecipitation at moderate pH_i_ values (4–10) and high pH_i_ ∼ 12. This latter scenario would agree with the findings of the NMR investigations that the Li sorption on goethite varies as a function of pH.^55,57^ Although we cannot completely determine which fractionation process dominates the Li uptake by goethite_LT_ with our data set, Rayleigh fractionation is favored because in this scenario (Figure 6b), two different α values, which suggest two different types of reactions, are respectively associated with pH_i_ values of 4–10 and pH_i_ ∼ 12. This scenario would be consistent with the observations of different Li uptake in the pH range from 4 to 10 compared to pH ∼ 12, as shown by the pH_f_ (Figure 4), Li uptake capacity (Figure 3), and NMR results.^57^ Also, in this case, the results of the goethite_LT_ experiments conducted at pH_i_ ∼ 12 in both Experiment 1 and 2 are consistent with each other, giving almost identical fractionation factors (Figure 6b,d).

Furthermore, we note that the calculated fractionation factor may be at the higher limits of the true value, as the goethite_LT_ particles have sizes (<100 nm; Figure 2) that are smaller than the pore size of the filter (0.2 μm), such that centrifugation at 4000 rpm may not be totally efficient at completely isolating the nanoparticles from the fluid. Hence, the fluid chemistry could be partly distorted toward lower δ^7^Li values (but also higher [Li]) by a potential mixture toward any isotopically light goethite_LT_ remaining in the analyzed solution. In addition, the magnitude of Li isotope fractionation observed in our experiments (−17 and −20‰) is significantly greater than that derived from the only previous experimental study that examined Li isotope fractionation during interactions with Fe-oxides (−3.5‰ for ferrihydrite, calculated using eqs 1 and 2).^34^ Finally, our results are comparable to the estimated values from acid-reductive leaching methods (−17 to −28‰),^52^ which implies that carefully operated leaching methods supported by measurements of trace element ratios in the leachates could be a valid approach to target the composition of Fe-oxide phases in natural samples.

In summary, dissolved Li can be taken up by poorly crystalline goethite_LT_ particles over a wide range of pH values from 4 to 12. This Li uptake is associated with a Li isotope fractionation of Δ^7^Li_oxide-fluid_ ∼ −17 to −20‰. Previous studies suggested that the Li adsorption is due to adsorption at inner-sphere sites at pH values greater than the PZC of goethite,^55,57^ and we further suggest that Li sorption through the dissolution–reprecipitation of active sites may also be an important process, especially under conditions where pH < 8, and should be investigated by future studies.

### 4.3 Mineral Crystalline State as an Often-Overlooked Factor Affecting Mineral–Water Interactions

An important finding from our study is that a given mineral can show distinctive geochemical behavior when in different crystalline states. Specifically, poorly crystalline goethite_LT_ can take up ∼90% of dissolved Li with a fractionation Δ^7^Li_oxide-fluid_ ∼ −20‰ in an alkaline solution at pH ∼ 12, whereas highly crystalline geothite_HT_ particles are not reactive with dissolved Li at pH ∼ 12 or over a wide range of pH conditions. To date, in the isotope geochemistry community, most fluid–rock interaction studies focus on the effect of mineralogy. Here, we argue that mineral crystallinity can also play an important role. It is well known that Fe-oxide minerals display various crystallinity states and can be relatively quickly recrystallized.^53,68,76,77^ In natural systems, it is therefore to be expected that well-aged, and hence more crystalline, Fe-oxides would not actively react with fluid Li. For aluminosilicate clays, this phenomenon of crystallinity affecting water–rock interaction has also been observed for Li adsorption onto laboratory-synthesized smectite.^10^ Specifically, Vigier et al.^10^ reported that hectorite synthesized at lower temperatures has a greater capacity for Li adsorption due to the presence of more crystal defects, in agreement with the geochemical behavior of Li observed in the present study. Additionally, the previously reported observation of the preferential release of ^6^Li during the dissolution of poorly crystalline kaolinite at low pH values^12^ can be further explained by the dissolution of octahedral structures at active sites.

Therefore, in both the case of clay minerals and Fe-oxides, the effect of the crystalline state needs to be considered, and here, we raise two related concerns. First, in experimental studies of water–rock interactions, mineralogy has often been addressed, whereas mineral crystallinity has rarely been examined. Therefore, directly applying sorption coefficients or isotope fractionation factors obtained from experimental studies to natural settings may introduce biases. Future studies should further investigate this under-addressed issue, potentially by studying secondary phases in both poorly crystallized and well-crystallized secondary phases, as well as studying amorphous phases. Second, mineral crystalline states vary between natural field areas. In kinetically limited weathering regimes (typically characterized by high physical erosion rates), particles have short residence times, so minerals tend to be less crystalline than in supply-limited weathering regimes (typically characterized by low physical erosion rates), where particle residence times are long. For example, goethite particles from Iceland, an example of a kinetically limited setting, are nanocrystalline,^78^ whereas goethite particles observed in laterite profiles from the Congo Basin, a typical supply limited environment, are well crystallized and can have lengths greater than 10 μm.^79^ According to our experimental results, these goethite particles exhibit different geochemical characteristics. Therefore, not only is it important to analyze the mineralogy using XRD techniques, but complementary observations of sample particles using electron-sourced imaging techniques (such as SEM and TEM) would greatly improve our knowledge of the coupled geochemical and mineralogical behavior, with implications for Li isotope characteristics in natural environments.

## 5 Implications and Conclusions

Our study provides new experimental constraints on the fundamental behavior of Li and Li isotopes during their interaction with Fe-oxides. First, we show that Li can be taken up by poorly crystalline goethite nanoparticles, resulting in Li isotope fractionation Δ^7^Li_oxide-fluid_ ranging from −17 to −20‰. The fractionation factor calculated from our experiments is important for improving our understanding of highly weathered soil profiles such as laterites, as well as in subsurface water–rock interactions where Fe-oxide formation can be common.^15,21,38,48,80,81^ Second, we show that the Li uptake by goethite is controlled by both the fluid pH and the goethite crystallinity. Poorly crystalline goethite can take up ∼90% dissolved Li at pH ∼ 12, likely through reprecipitation reactions occurring at active sites, and a significant fraction of the Li uptake could be released with extraction under lower pH conditions. In contrast, Li adsorption by outer-sphere complexation at the surfaces of well-crystalline Fe-oxides appears to be insignificant.

These results have two significant implications. To an extent, the Li uptake and Li isotope fractionation associated with neoformation at mineral surfaces could be at least partially responsible for Li isotope signals observed in floodplains.^4,73^ For example, when poorly crystalline materials formed in upper catchment areas are transported and deposited in lower floodplains, water–rock interactions with these materials can further modify the fluid Li chemistry through adsorption, incorporation, and isotope fractionation. Similarly, at the land–sea interface, where seawater generally has higher pH values than river waters, the interaction of poorly crystalline detrital materials with seawater could occur during sediment transport into the mixing zone or during sea-level rise over longer time scales.^82^ These effects could potentially be considered by re-examining observations made in estuaries.^16,83,84^ Furthermore, our results point to the potential of poorly crystalline goethite for efficient large-scale industrial extraction of Li, which warrants further investigation because Li is in high demand for the energy transition.^85^

Finally, we demonstrate that constraining sorption behavior during water–rock interactions requires the effects of mineral crystallinity to be evaluated. Hence, we suggest that (1) a better understanding of crystal nucleation, growth, and defect recrystallization should be an important target for future studies; and (2) future studies should prioritize further characterization of nanoparticles in combination with quantification of fluid chemistry with suitable methods.

## Data Availability

For the purpose of open access, the author has applied a “Creative Commons Attribution (CC BY) license” to any author accepted manuscript version arising. The original XRD, ATR-FTIR, and Raman results are freely available at Utrecht University Yoda data repository: 10.24416/UU01-MYX8OZ

## Supporting Information Available

Solid characterization of Fe-oxide particles used in the sorption experiments (XRD, ATR-FTIR, and Raman); thermodynamic calculations of fluid chemistry; and calculation of Li isotope fractionation during sorption onto poorly crystalline goethite (PDF)
