# Supplementary material for: Coupling of Li–Fe: Li Isotope Fractionation during Sorption onto Fe-Oxides
Source: ACS Earth Space Chem. 2024 Nov 25;9(1):49–63. doi: 10.1021/acsearthspacechem.4c00205 (PMC11744929; doi:10.1021/acsearthspacechem.4c00205)
Supplement: Supplementary file 1 — sp4c00205_si_001.pdf [file sp4c00205_si_001.pdf]

Supplementary Materials

Manuscript title: Coupling of Li-Fe: Li isotope fractionation during sorption onto Fe-oxides

Authors: Xu (Yvon) Zhang[a*], David J. Wilson[b], Maartje F. Hamers[a], Philip A. E. Pogge von Strandmann[c], Oliver Plümper[a], Helen E. King[a]

[a]Department of Earth Sciences, Utrecht University, 3584 CB Utrecht, The Netherlands
[b]LOGIC, Department of Earth Sciences, University College London, WC1E 6BS London, United Kingdom
[c]MIGHTY, Institute for Geosciences, Johannes Gutenberg University Mainz, D-55128 Mainz, Germany

Journal: *ACS Earth and Space Chemistry*

Supporting figures: 7
Fig S1-S5: Solid characterization of Fe-oxide particles used in the sorption experiments (XRD, ATR-FTIR, and Raman)
Fig S6: Thermodynamic calculations of fluid chemistry
Fig S7: Modelling of Li isotope fractionation

Supporting tables: 1
Table S1: Calculated Li isotope fractionation and the associated fractionation factors for Li sorption onto poorly crystalline goethite

Number of pages: 10

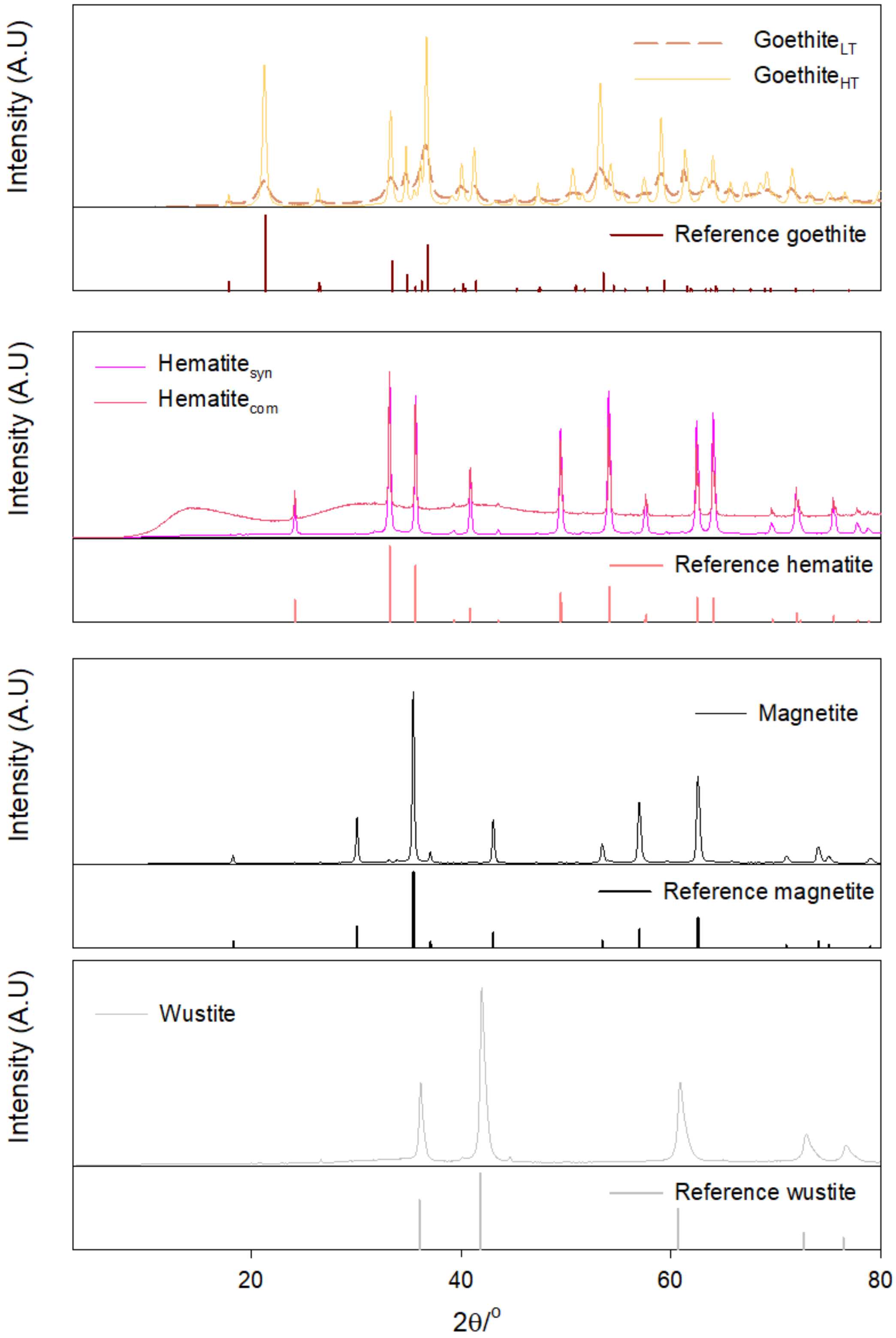

Figure S1 XRD patterns of the Fe-oxide particles used for Li sorption experiments. The reference data for goethite[1], hematite[2], magnetite[3], and wüstite[4] are from the American Mineralogist Crystal Structure Database[5].

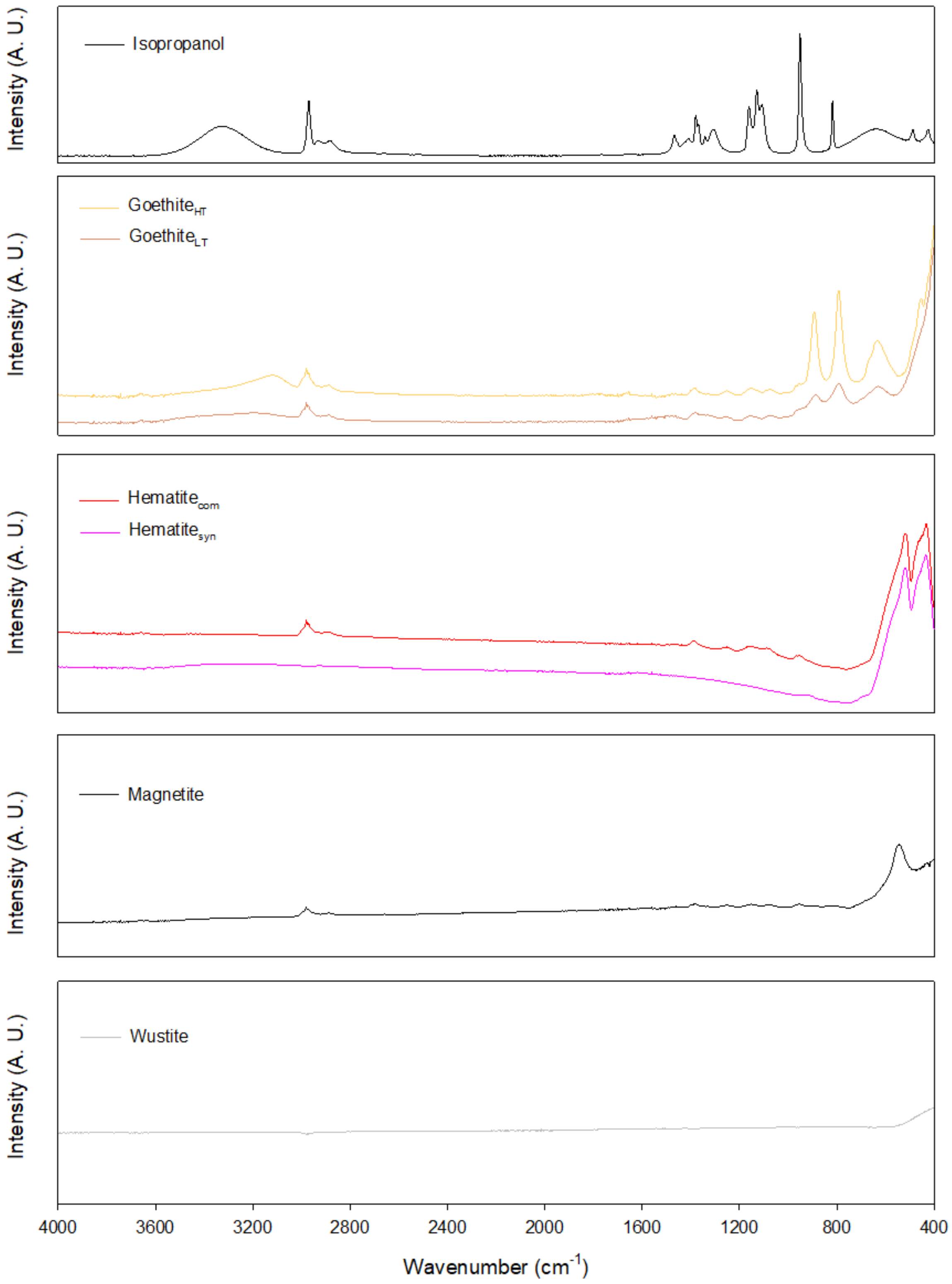

Figure S2 FTIR absorbance spectra of the isopropanol used for instrumental cleaning and the Fe-oxide particles used for Li sorption experiments.

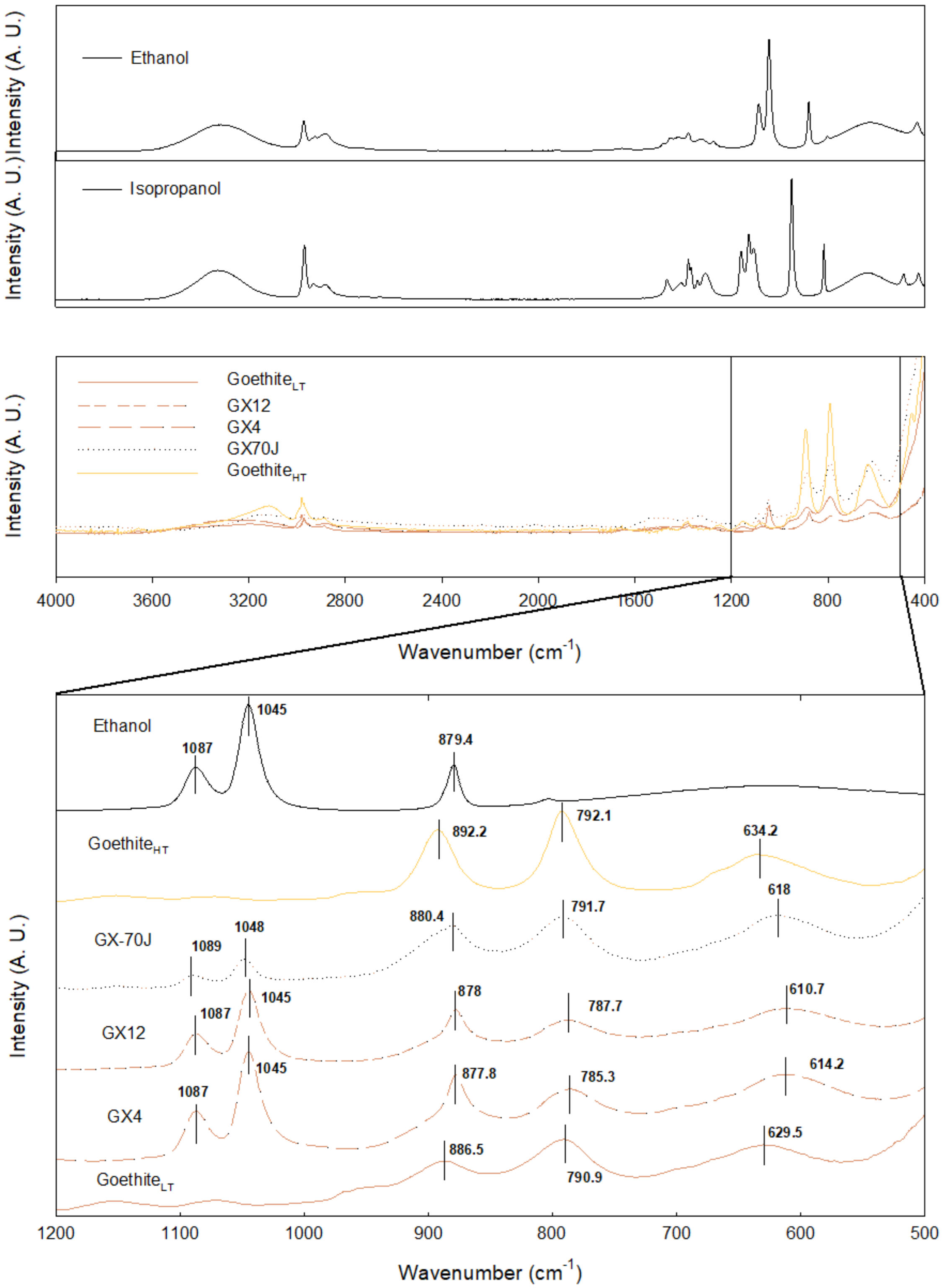

Figure S3 FTIR absorbance spectra of ethanol (used during Fe-oxide centrifugation/filtration), isopropanol (used for ATR-FTIR instrumental cleaning), and selected goethite particles, including unreacted goethite$_{HT}$ particles, unreacted goethite$_{LT}$ particles, goethite particles recovered from two sub-experiments in Experiment 1 (GX4 and GX12), and goethite particles recovered from Experiment 2 (GX-70J).

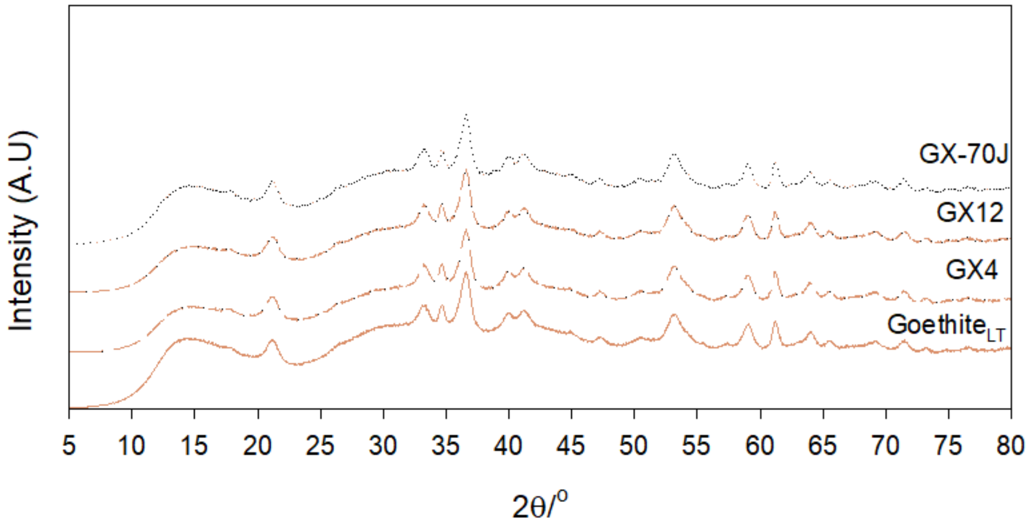

Figure S4 XRD patterns of selected goethite$_{LT}$ particles, including unreacted goethite$_{LT}$, goethite particles recovered from two sub-experiments in Experiment 1 (GX4 and GX12), and goethite particles recovered from Experiment 2 (GX-70J).

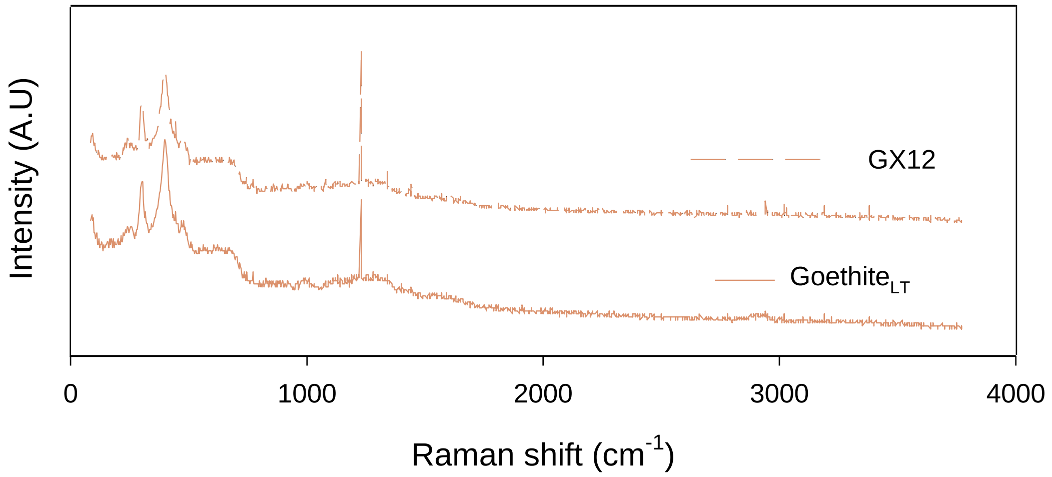

Figure S5 Raman spectra of unreacted goethite$_{LT}$ particles and goethite particles recovered from sub-experiment at pH~12 in Experiment 1 (GX12).

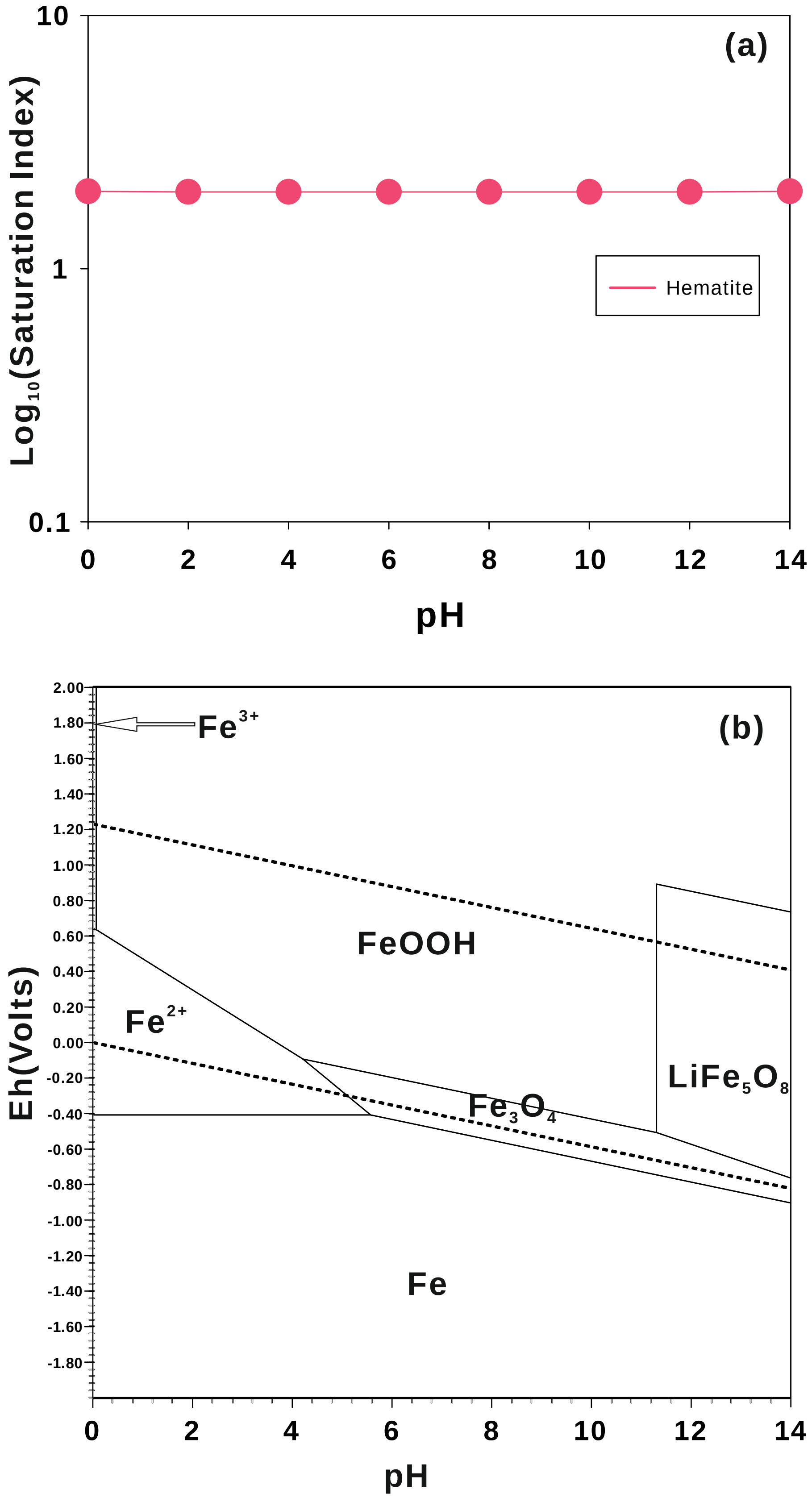

Figure S6 Thermodynamic calculations of (a) fluid saturation index in Experiment 1 using Phreeqc, and (b) Pourbaix diagram for the Li-Fe fluid system using HSC chemistry version 9.0.

Phreeqc calculation inputs:

```
EQUILIBRIUM_PHASES 1
    Goethite  0 10

SOLUTION 1-100
    temp      25
    pH        X (X=0, 2, 4, 6, 8, 10, 12, and 14)
    pe        4
    redox     pe
    units     mmol/kgw
    density   1
    Na        0.1 mol/kgw
    Cl        0.1 mol/kgw
    Li        0.2 mmol/kgw
    water     1 # kg
```

SI calculation outputs:

pH	SI (Hematite)
0	2.02
2	2.01
4	2.01
6	2.01
8	2.01
10	2.01
12	2.01
14	2.02

Pourbaix diagram calculation inputs by HSC Chemistry version 9.0:

Temperature 25°C, total Fe 1 M, total Li 1 M

Outputs

Fe		Fe^{2+}		FeOOH		Fe_3O_4		$LiFe_5O_8$		Fe^{3+}	
pH	Eh (Volts)	pH	Eh (Volts)	pH	Eh (Volts)	pH	Eh (Volts)	pH	Eh (Volts)	pH	Eh (Volts)
0	-0.409	0	0.638	14	0.737	14	-0.762	14	0.737	0.079	2
5.569	-0.409	0.079	0.638	11.31	0.895	11.31	-0.509	11.31	0.895	0.079	1.687
14	-0.904	4.224	-0.093	11.31	-0.509	4.224	-0.093	11.31	-0.509	0.079	0.638
14	-2	5.569	-0.409	4.224	-0.093	5.569	-0.409	14	-0.762	0	0.638
0	-2	0	-0.409	0.079	0.638	14	-0.904			0	2
				0.079	1.687						
				0.079	2						
				14	2						

Table S1 Calculated Li isotope fractionation and the associated fractionation factors in Experiments 1 and 2 for Li sorption onto poorly crystalline goethite

Sample	Final δ^7Li in solution (‰)	δ^7Li_{ad} (‰)	$\Delta^7Li_{oxide-fluid}$ (‰)	α (equilibrium fractionation)	α (Rayleigh fractionation)
**Experiment 1**					
GX4	16.6	0.6	-16.0	0.9841	0.9860
*GX6**	*13.7*	*9.8*	*-3.9*	*0.9961*	*0.9966*
GX8	16.9	-0.2	-17.1	0.9830	0.9850
GX10	16.8	0.2	-16.6	0.9836	0.9860
GX12	28.1	10.9	-17.2	0.9829	0.9930
**Experiment 2**					
D0	12.8				
D1	32.1	11.2	-20.9		
D2	32.1	11.3	-20.8		
D4	32.6	11.5	-21.1	0.9801	0.9933
D8	32.1	11.6	-20.5		
D16	31.8	11.7	-20.1		
D30	31.3	11.8	-19.5		
D70	29.7	11.8	-17.9		

*Considered as a possible artifact

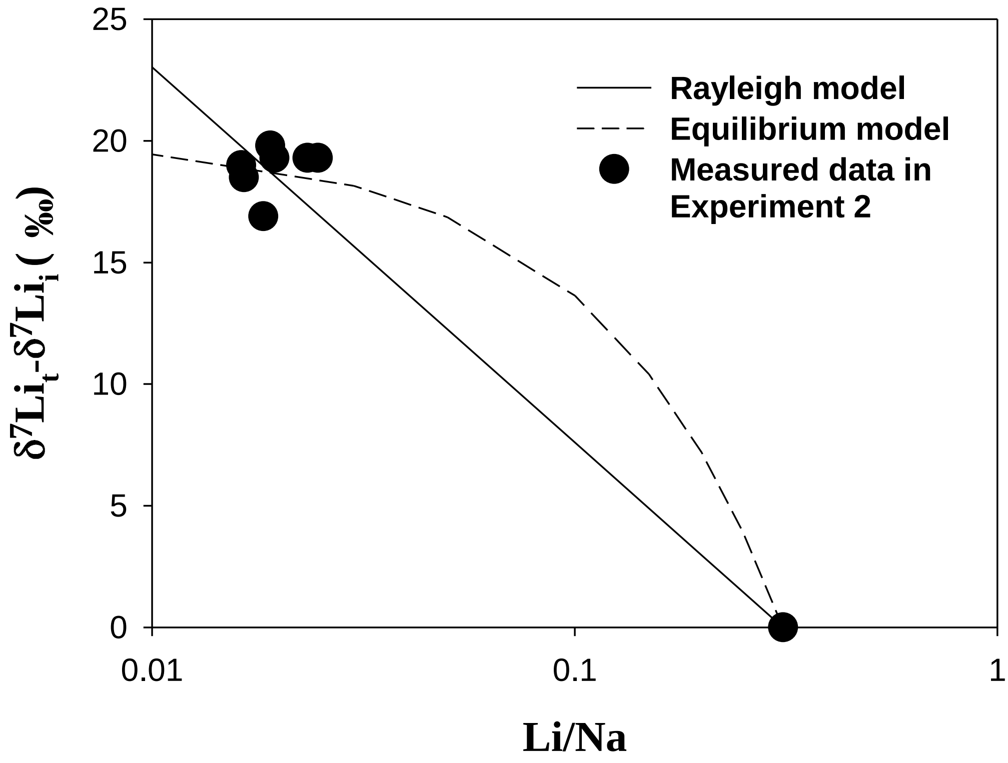

Figure S7 Changes in fluid δ^7Li values, expressed as the difference between δ^7Li values at the sampling point (δ^7Li_t) and the initial value (δ^7Li_i), as a function of Li/Na ratios (logarithmic scale) during Li uptake by $goethite_{LT}$.

Supplementary References:

(1) Gualtieri, A. F.; Venturelli, P. In Situ Study of the Goethite-Hematite Phase Transformation by Real Time Synchrotron Powder Diffraction. *American Mineralogist* **1999**, *84* (5–6), 895–904.

(2) Maslen, E. N.; Streltsov, V. A.; Streltsova, N. R.; Ishizawa, N. Synchrotron X-Ray Study of the Electron Density in α-Fe 2O 3. *Acta Crystallographica Section B* **1994**, *50* (4), 435–441.

(3) Haavik, C.; Stølen, S.; Fjellvåg, H.; Hanfland, M.; Häusermann, D. Equation of State of Magnetite and Its High-Pressure Modification: Thermodynamics of the Fe-O System at High Pressure. *American Mineralogist* **2000**, *85* (3–4), 514–523.

(4) Katsura, T.; Iwasaki, B.; Kimura, S.; Akimoto, S. High- Pressure Synthesis of the Stoichiometric Compound FeO. *J Chem Phys* **1967**, *47* (11), 4559–4560.

(5) Downs, R. T.; Hall-Wallace, M. The American Mineralogist Crystal Structure Database. *American Mineralogist* **2003**, *88* (1), 247–250.
